# Commentary: “Prolactinomas: Prognostic Factors of Early Remission After Transsphenoidal Surgery”

**DOI:** 10.3389/fendo.2021.695498

**Published:** 2021-05-14

**Authors:** Lukas Andereggen, Emanuel Christ

**Affiliations:** ^1^ Department of Neurosurgery, Kantonsspital Aarau, Aarau, Switzerland; ^2^ Faculty of Medicine, University of Bern, Bern, Switzerland; ^3^ Department of Endocrinology, Diabetology and Metabolism, University Hospital of Basel, Basel, Switzerland

**Keywords:** dopamine agonists, follow-up, Knosp grading, microadenoma, macroadenoma, prolactinoma, remission, surgery

We read with great interest the article by Zielinski et al. (1) reporting indicators for early remission (i.e., 3 months) after transsphenoidal surgery in 48 prolactinoma patients resistant to or intolerant of dopamine agonists (DAs). The only independent predictor for early remission was missing cavernous sinus invasion (p<0.001) (1), for which radiological assessment of Knosp grades is advised. The Knosp classification is used to describe invasiveness of an adenoma into the cavernous sinus (2, 3). In grade 0, the adenoma does not extend the medial carotid line; in grade 1, the adenoma extends to the medial carotid line; in grade 2, the adenoma extends beyond the median carotid line; in grade 3, the adenoma extends to the lateral carotid line; and in grade 4, the internal carotid artery is completely surrounded by the adenoma (2). The higher the grading, the more difficult complete surgical removal can be attained (2). In surgical series, the Knosp grading not only plays an important role in controlling hyperprolactinemia in patients with DA resistance or intolerance (4). Likewise, in patients with a surgical first approach, missing cavernous sinus invasion (i.e., Knosp grade ≤1) is pivotal to attain high remission rates along with the avoidance of ongoing DA therapy (5–7).

Significant predictors of DA resistance have been identified as male gender, large adenomas, and prolonged time to prolactin (PRL) normalization (8, 9). In contrast to the patient cohort included in Zielinski et al.’s study (1), many patients with evidence of DA resistance or intolerance are thus men and present with larger prolactinomas and/or adenomas with invasion of the cavernous sinus (i.e., Knosp grade ≥2) (10–12). Namely, in women, amenorrhea is investigated early on whereas men with hypogonadism often suffer from more nonspecific symptoms, subsequently presenting at older age with larger and/or invasive adenomas at the time of diagnosis (12–14). Thus, it has been proposed that surgery be discussed in men because of a higher likelihood of DA resistance and aggressive behavior of prolactinoma (15), although men with prolactinomas usually respond to medical treatment with no need for any additional treatment (16). In Zielinski et al.’s reported cohort, the majority of patients harbored a microadenoma or adenoma with a Knosp grade of ≤1 (1). In this selected patient cohort, however, we consider that a primary surgical approach might at least be interdisciplinary discussed as an alternative to DA-agonist therapy. There is a good chance of non-dependency on DA therapy in dedicated pituitary centers using a surgical-first approach in patients with adenomas not infiltrating the cavernous sinus (i.e., Knosp grade ≤1) (7). Namely, after a median follow-up of ≈7 years, we noted that DA therapy was ultimately required in 24% of patients with microprolactinomas, compared to 49% with macroprolactinomas (p = 0.03). As for the latter, DA was required in 76% with Knosp grade 1 compared to 29% with Knosp grade 0 macroprolactinomas (p = 0.004) (7).

Furthermore, prior DA therapy may induce adenoma fibrosis, potentially hampering surgical remission rates (17, 18). While cabergoline has a better drug tolerance profile and allows for easier administration thanks to its longer half-life (19–21), it is the preferred drug when it comes to medical treatment of prolactinomas (22). It has been reported that prolactinomas exposed to bromocriptine may develop tumor fibrosis (18, 23–29), yet rarely after cabergoline (18, 30). Menucci et al. reported that patients exposed to bromocriptine are more likely to have tumor fibrosis than patients that are untreated or treated with cabergoline alone (18). Namely, in 21 prolactinoma patients with DA therapy, adenoma fibrosis was noted in 83% when treated with bromocriptine, and in 22% of patients treated with cabergoline, with a statistically significant difference in patients exposed to bromocriptine for at least one month (p< 0.05) (18). Given that bromocriptine was prescribed in 75% of all patients in the Zielinski study cohort (1), and that a Knosp grade 0 and 1 was noted in 80% of patients (1), this is a strong argument that in this selected cohort a first line approach might have been a valuable treatment option, in particular as tumor fibrosis hampers complete adenoma resection, especially after bromocriptine therapy (18, 23–29).

Besides, in prolactinoma patients, the assessment of long-term outcomes is pivotal. As DAs can be tapered 24 months after initiation of medical therapy when prolactin (PRL) levels are normalized (31), early recurrence of hyperprolactinemia following discontinuation of DAs have been reported, particularly in patients with macroprolactinomas (32–35). However, macroprolactinomas per se do not impede low remission rates, but rather does their extension into the cavernous sinus (7). As for surgical series, recurrences are observed in one-third of prolactinoma patients and may occur as late as 13 years after surgery (10). It has been shown that hyperprolactinemia recurs early in most macroprolactinomas (93%) and microprolactinomas (64%) following DA therapy discontinuation, while cessation of DA cannot be recommended even after 7 years of therapy (36). A recent meta-analysis, however, showed that long-term remission was lower after DA withdrawal (34%) than after transsphenoidal surgery (64%) (37), while a previous meta-analysis reported even lower (21%) long-term remission rates after DA withdrawal (38). While we noted that recurrence-free intervals were significantly shorter in patients with a Knosp grade 1 adenoma (110.5 ± 32.2 months) than in those with a Knosp grade 0 adenoma (365.4 ± 22.9 months; log-rank test, p<0.001), recurrence-free intervals did not differ significantly with regard to adenoma size (7). In this regard, reporting the number of patients who remain off medication is an important outcome measure, as surgery is a known effective alternative treatment option in patients who are intolerant or resistant to medical therapy (39).

Furthermore, the long-term sequelae of cumulative DA doses might become an argument for surgery in younger patients (15, 40–43). Although side effects of DAs are infrequently encountered, Ono and colleagues reported that up to 18% of patients needed persistent high-dose cabergoline treatment in order to normalize hyperprolactinemia, irrespective of prolactinoma size (44). In this regard, cumulative doses of DA might account for long-term adverse effects (40–42), and new concerns about long-term safety of DAs have emerged over time in multiple studies (15, 41, 45, 46). DAs have been associated with side effects such as nausea, dizziness, and postural hypotension (20). Although side effects of cabergoline were recorded in 68% of women in a large cohort with hyperprolactinemic amenorrhea, only 3% of them ultimately had to discontinue drug therapy due to intolerance (19). Also, cabergoline-associated valvulopathy is uncommon (47, 48), and its clinical significance remains unclear (49). However, with regard to persistent DA therapy in the long term, cumulative DA doses might become an argument for surgery in younger patients (15, 40–43). In addition, personality changes associated with DAs have been reported, including gambling, hypersexuality and compulsive shopping (41, 46, 50). It is possible that patients do not mention these effects due to feelings of shame, with potential detrimental psychosocial consequences (45). In a large study by Bancos and coworkers in patients with prolactinomas with ongoing or past DA therapy compared to patients with non-functioning pituitary adenomas (NFPA) without DA therapy, the prevalence of impulse control disorders was significantly higher in prolactinoma patients (24.6% vs. 17.14%), or with regard to the general population (8.4%) (41). Thereby, men with prolactinomas treated with DAs showed a 9.9 higher risk than did men with NFPA (41). Dogansen suggested that because impulse control disorders may be prevalent in one out of six patients with prolactinoma treated with DAs, endocrinology specialists should be particularly attentive to male patients with a history of addictive behavior (51).

While giant invasive prolactinomas remain a therapeutic challenge (52–54), and DA is indicated as the primary therapy in them (55), including giant prolactinomas that cause visual field deficits (56), interdisciplinary consensus findings, along with careful MRI evaluation, are advised when it comes to the primary treatment choice in prolactinoma patients (5, 6, 35, 57). Whether transsphenoidal surgery in prolactinomas is superior to standard care as a first-line approach or a second-line treatment has to be investigated, and we will follow with interest the results of the PRolaCT trial (NCT 04107480).

## Author Contributions

LA designed and wrote the article. EC contributed to the study design and critically revised the commentary. All authors contributed to the article and approved the submitted version.

## Conflict of Interest

The authors declare that the research was conducted in the absence of any commercial or financial relationships that could be construed as a potential conflict of interest.
